# Multiple sclerosis plasma IgG aggregates induce complement-dependent neuronal apoptosis

**DOI:** 10.1038/s41419-023-05783-3

**Published:** 2023-04-08

**Authors:** Wenbo Zhou, Michael Graner, Petr Paucek, Cheryl Beseler, Matthew Boisen, Andrew Bubak, Francisco Asturias, Woro George, Arin Graner, David Ormond, Timothy Vollmer, Enrique Alvarez, Xiaoli Yu

**Affiliations:** 1 Department of Neurosurgery, University of Colorado Anschutz Medical Campus, Aurora, Colorado, 80045 USA; 2 Department of Environmental, Agricultural and Occupational Health, University of Nebraska Medical Center, Omaha, NE 68198 USA; 3 Zalgen Labs, LLC, 12635 E. Montview Blvd., Suite 131, Aurora, Colorado, 80045 USA; 4 Department of Neurology, University of Colorado Anschutz Medical Campus, Aurora, Colorado, 80045 USA; 5 Department of Biochemistry and Molecular Genetics, University of Colorado Anschutz Medical Campus, Aurora, Colorado, 80045 USA

**Keywords:** Cell death in the nervous system, Multiple sclerosis, Cellular neuroscience, Diagnostic markers

## Abstract

Grey matter pathology is central to the progression of multiple sclerosis (MS). We discovered that MS plasma immunoglobulin G (IgG) antibodies, mainly IgG1, form large aggregates (>100 nm) which are retained in the flow-through after binding to Protein A. Utilizing an annexin V live-cell apoptosis detection assay, we demonstrated six times higher levels of neuronal apoptosis induced by MS plasma IgG aggregates (*n* = 190, from two cohorts) compared to other neurological disorders (*n* = 116) and healthy donors (*n* = 44). MS IgG aggregate-mediated, complement-dependent neuronal apoptosis was evaluated in multiple model systems including primary human neurons, primary human astrocytes, neuroblastoma SH-SY5Y cells, and newborn mouse brain slices. Immunocytochemistry revealed the co-deposition of IgG, early and late complement activation products (C1q, C3b, and membrane attack complex C5b9), as well as active caspase 3 in treated neuronal cells. Furthermore, we found that MS plasma cytotoxic antibodies are not present in Protein G flow-through, nor in the paired plasma. The neuronal apoptosis can be inhibited by IgG depletion, disruption of IgG aggregates, pan-caspase inhibitor, and is completely abolished by digestion with IgG-cleaving enzyme IdeS. Transmission electron microscopy and nanoparticle tracking analysis revealed the sizes of MS IgG aggregates are greater than 100 nm. Our data support the pathological role of MS IgG antibodies and corroborate their connection to complement activation and axonal damage, suggesting that apoptosis may be a mechanism of neurodegeneration in MS.

## Introduction

Pathology of the grey matter, including the cortex, is central to the progression of multiple sclerosis (MS) [1], a neuroinflammatory demyelinating disease with neurodegeneration at chronic stages [2]. Axonal injury correlates with demyelination [3], and grey matter pathology correlates with physical disabilities and cognitive impairment in MS [4–7]. Neuronal vulnerability is relevant to neurodegeneration and lesion progression, with neuronal death being prominent at the earliest stages of the disease [8, 9].

Over 20 times more immunoglobulin G (IgG) can be extracted from MS plaques than those from the control brain [10]. The common features of acute demyelinating plaques are marked deposition of IgG and activated complement [11–13] which are consistently present in active lesions and cortical grey matter lesions [14, 15]. Demyelination and axonal damage occur in the presence of antibodies and require activation of the entire complement cascade [3]. Recently, neuron-specific activation of necroptosis signaling in MS cortical grey matter was demonstrated [16]; complement activation was detected in clinically distinct murine models of MS [17], and complement component 1q (C1q) was shown to be a critical mediator for microglia in MS lesions [18].

MS IgG oligoclonal bands (OCB) are associated with disease activity, disability, and brain atrophy [19–22]. Soluble factors in cerebrospinal fluid (CSF) and supernatants of MS B cell cultures induced axonal damage and neuronal apoptosis [23, 24]. However, whether these soluble factors are IgG antibodies have not been demonstrated; thus the pathological role of IgG antibodies in MS remains controversial [25].

Herein, we provide evidence that MS plasma IgG antibodies form large aggregates (>100 nm), inducing complement-dependent apoptosis in neuronal cells. We characterized the IgG aggregates by nanoparticle tracking analysis and transmission electron microscopy; we utilized multiple cell models and evaluated the cytotoxicity of the IgG aggregates from 190 MS and 160 control plasma samples. Our data support the pathological role of MS IgG antibodies and may provide strategies for novel therapeutics, especially for progressive MS where effective therapeutics are lacking.

## Results

### Three times more IgG1 were detected in MS plasma Protein A flow-through than in healthy controls

**Fig. 1:**
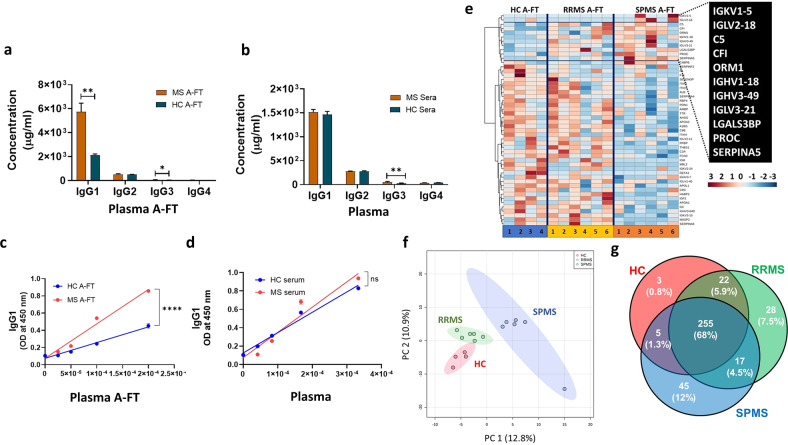
Significantly higher levels of IgG1 and IgG3 antibodies are detected in the flow-through after Protein A incubation with MS plasma. **a** Significantly higher levels of IgG1 and IgG3 were detected in MS A-FT (*n* = 7) compared to HC A-FT (*n* = 4) (*p* = 0.0061 for IgG1, *p* = 0.04 for IgG3, Mann Whitney test). **b** No differences in IgG1 but higher levels of IgG3 were found in MS plasma compared to HC (*p* = 0.0061). **c** IgG1 dose-response ELISA confirmed the presence of higher levels of IgG1 in MS A-FT compared to HC A-FT. **d** No difference in IgG1 was detected in the original MS plasma compared to HC plasma. **e** Heatmap of Mass Spec proteomic analyses revealed that MS A-FT has higher levels of unique protein sets consisting of IgG heavy and light chains and complement components (highlighted in the black box). SPMS = 6, RRMS = 6, HC = 4. **f** Principal component analysis shows that clear distinctions between SPMS, RRMS, and HC are observed based on proteomics of plasma A-FT. **g** Venn diagram showing the number of proteins identified in each group.

Higher levels of plasma immunoglobulin G3 (IgG3) have been shown to predict the development of MS from clinically isolated syndrome [26]. We recently reported that increased levels of IgG3 in plasma, but not in CSF, can distinguish MS from other neurological disorders (OND) [27]. To investigate whether enriched plasma IgG3 could be a biomarker for MS, we utilized the unique feature of IgG3 which does not bind Protein A [28]. We collected the flow-through/unbound portions after incubation of plasma in Protein A-coated plates which are named as “Protein A flow-through (A-FT)”. We determined the IgG subclass levels in the A-FT samples from 7 secondary progressive MS (SPMS) patients and 7 healthy controls (HC) using human IgG subclass enzyme-linked immunosorbent assay (ELISA) kits. We discovered that SPMS plasma A-FT contained nearly three times more IgG1 (*p* = 0.0061) and two times more IgG3 (*p* = 0.04) compared to HC A-FT (Fig. 1a). However, no such difference in IgG1 was detected in the neat SPMS plasma; higher IgG3 levels in MS plasma were also found (Fig. 1b) which is consistent with our previous report [29]. No differences in IgG2 and IgG4 in both A-FT and original plasma were detected (Fig. 1a, b). Dose-response ELISA further confirmed the presence of significantly higher levels of IgG1 in MS A-FT (Fig. 1c) only, but not in paired MS plasma (Fig. 1d). We carried out Mass Spectrometry proteomic analyses using A-FT samples from HC, Relapsing Remitting MS (RRMS), and SPMS. A heatmap revealed that the unique proteins present in MS A-FT consisted of IgG heavy and light chains and complement components (Fig. 1e). The principal component analysis demonstrated that the proteins between SPMS, RRMS, and HC in the A-FT are distinctive (Fig. 1f). Further analysis revealed higher total numbers of proteins in both RRMS and SPMS A-FTs (322) compared to HC A-FT (285), and unique proteins in RRMS (7.5%) and SPMS (12%), in contrast to HC A-FT which had only 3 unique proteins (0.8%). Specifically, IgG heavy chains IGHV1–3, IGHV3–43, and IGHV3–49 are unique for RRMS and SPMS, but not present in HC A-FT (Fig. 1g, Supplementary Figs. 1 and 2).

### MS plasma A-FT induces complement-dependent cytotoxicity in neuronal cells

**Fig. 2:**
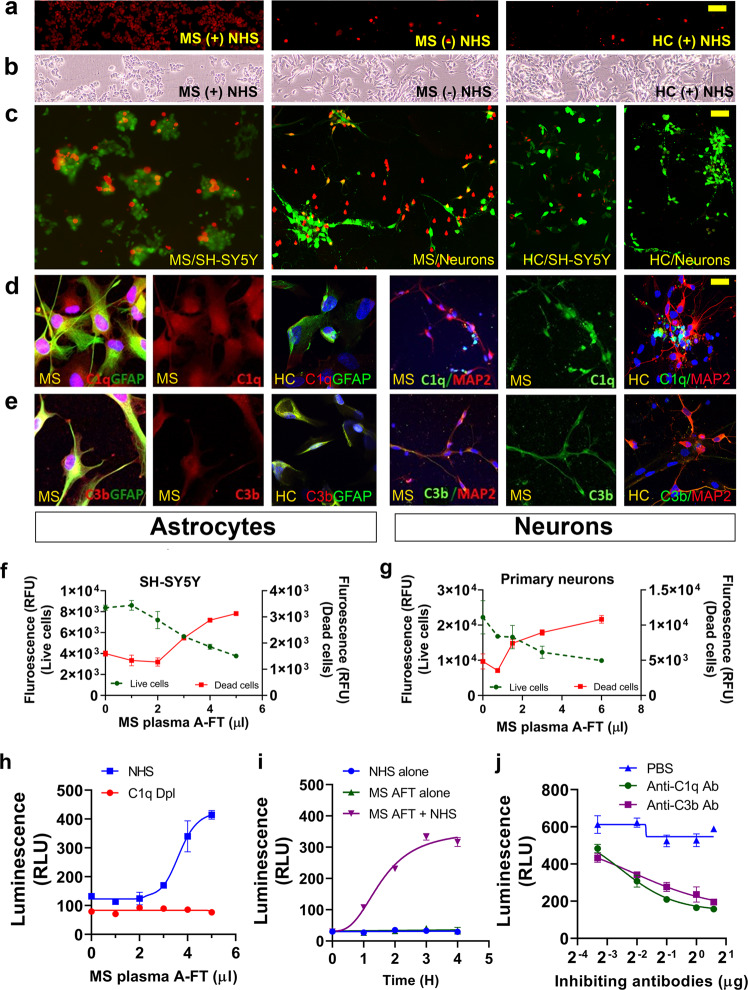
MS plasma A-FT induces complement activation and neuronal cell death. **a**, **b** Normal human serum (NHS) is required for MS A-FT cytotoxicity. SH-SY5Y cells were treated with MS or HC A-FT for 4 h in the presence of propidium iodide followed by live-cell imaging. Fluorescent (**a**, left panel) and phase contrast imaging (**b**, left panel) showed a massive number of dead cells in wells incubated with MS A-FT plus NHS, and few dead cells in wells without NHS [MS (-) NHS] (middle panels). HC A-FT treated cells [HC (+) NHS] revealed few dead cells (right panels). Scale bar, 100 µm. **c** LIVE/DEAD™ Viability/Cytotoxicity assay demonstrates neuronal damage caused by MS plasma A-FT, but not by HC plasma A-FT, in SH-SY5Y and primary human neurons. Viable cells are shown in green and dying and dead cells are in red. Scale bar, 50 µm. **d**, **e** Confocal microscopy demonstrates the presence of complement activation components C1q and C3b in astrocytes and neurons. Cells were treated with MS A-FT for 2 h, followed by double immunostaining for C1q and C3b and cell markers (GFAP for astrocytes and MAP2 for neurons). Scale bar, 20 µm. **f**, **g** Dose-response curves of total live and dead cells treated with MS A-FT for 4 h in SH-SY5Y cells (**f**) and human primary neurons (**g**) overnight. **h** Incubation of MS A-FT with C1q-depleted serum (C1q Dpl) produced no cytotoxicity in SH-SY5Y cells. **i** Treatment of NHS alone or MS A-FT alone did not induce apoptosis; only MS A-FT plus NHS caused neuronal death. **j** MS A-FT-produced apoptosis was inhibited by anti-C1q and anti-C3b antibodies in a dose-dependent manner. The apoptotic cell death was measured by luminescence after 4 h of treatment.

We evaluated the cytotoxicity effects of MS A-FT in SH-SY5Y cells (as neuronal surrogates), human primary neurons, and human primary astrocytes (normal human astrocytes, NHA). We used pooled SPMS (*n* = 16) and pooled healthy control (HC) A-FT (*n* = 16) or randomly picked individual samples for cytotoxicity assays. Using propidium iodide (PI) to label membrane-permeant (dead) cells, we discovered that treating cells with MS A-FT plus normal human serum (NHS, as a complement source) for 4 h induced distinguishable cell death. However, few dead cells were observed with MS A-FT but without NHS or with HC A-FT treated cells (Fig. 2a, b), suggesting that MS A-FT causes cell death in a complement-dependent manner. Using LIVE/DEAD™ Viability/Cytotoxicity assay, we showed that apparent neuron death occurred after 30 min of incubation with MS A-FT in both SH-SY5Y cells and neurons, but not in HC A-FT treated neurons. Representative fluorescent images are shown as live (green) and dead cells (red) (Fig. 2c). To confirm that cell death is due to complement activation, we carried out immunocytochemistry with MS A-FT treated neurons and astrocytes. We demonstrated the presence of early complement activation components C1q and C3b. Evident staining of C1q (Fig. 2d) and C3b (Fig. 2e) are present in the cytoplasm of both cell types. In HC A-FT treated cultures, there were relatively few C1q and C3b positive cells (Fig. 2d, e). We further quantified cell death in MS A-FT treated neurons using live/dead assays from data reflective of the images in Fig. 2c. Dose-response curves of neuroblastoma/SH-SY5Y cells (Fig. 2f) and primary neurons (Fig. 2g) demonstrated a higher number of dead cells and a lower number of viable cells after MS A-FT treatment. We applied RealTime-Glo™ Annexin V Apoptosis Assay to assess apoptosis by measuring the real-time exposure of phosphatidylserine via luminescence signals. MS A-FT induced strong apoptosis in neuroblastoma (Fig. 2h, i). To definitively confirm if C1q is required for MS A-FT induced cytotoxicity, we showed that neurons incubated with MS A-FT plus C1q-depleted human serum (C1q Dpl, Fig. 2h) or NHS alone (Fig. 2i) did not undergo apoptosis, suggesting that the classical complement pathway is involved in the neuronal apoptosis. Additional data showed that incubation of anti-C1q and anti-C3b antibodies with MS A-FT inhibited neuronal apoptosis in a dose-dependent manner (Fig. 2j).

### Neuronal apoptosis induced by MS A-FT depends on IgG antibodies

**Fig. 3:**
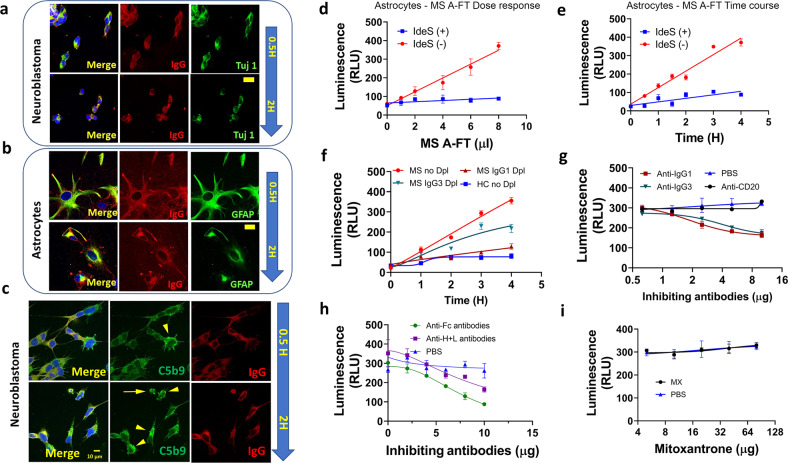
IgG antibody-mediated, complement-dependent neuronal apoptosis by MS A-FT. **a**, **b** Time course of confocal microscopy showing the presence of IgG antibodies in neuroblastoma (**a**) and astrocytes (**b**) treated with MS A-FT. IgG positive staining was observed after 30 min treatment and cell damage/death was more prominent after 2 h in both cells. Both IgG and cell markers were shown in the membrane-compromised/dying cells in a time-dependent manner. Scale bars: 20 µm (**a**), 10 µm (**b**). **c** Time course of immunostaining of complement membrane attack complex C5b9 and IgG. C5b9 (green) was co-localized with IgG (red). Arrowheads indicate C5b9 positive cells, and the arrow shows the apoptotic cell body. Scale bar, 10 µm. **d**, **e** The IgG-degrading enzyme IdeS abolishes apoptosis by MS A-FT in a dose- (**d**) and time-dependent manner (**e**)**. f** IgG1- and IgG3-depleted MS A-FT lost cell-killing capacity. IgG1 depletion reduced levels of apoptosis which nearly reaches the background levels as with HC A-FT. IgG3 depletion also showed a reduction of apoptosis. **g** Incubation with anti-IgG1 and anti-IgG3 antibodies reduced cell death in a dose-dependent manner, but MS therapeutics anti-CD20 antibodies did not. **h** MS A-FT-induced apoptosis is inhibited by anti-IgG antibodies (anti-Fc and anti- H + L). Notice that anti-Fc antibodies reduced more cell death than anti-H + L. **i** The current MS disease-modifying drug Mitoxantrone (MX) did not prevent A-FT-induced apoptosis.

We demonstrated the presence of IgG antibodies in damaged neuroblastoma and astrocytes incubated with MS A-FT using confocal immunocytochemistry (Fig. 3a, b). In both cell types, IgG antibodies were detected in the cytoplasm after 30 min incubation. Cell damage and cell death were more prominent at 2 h treatments, showing apoptotic nuclei, whereas the cytoplasm showed necrotic degeneration and progressive lysis of both nuclei and cytoplasm. Cells treated with HC A-FT showed almost no staining for IgG (Supplementary Fig. 3). Furthermore, we showed the co-localization of the complement membrane attack complex C5b9 and IgG in SH-SY5Y at 30 min and 2 h time points (Fig. 3c, Supplementary Fig. 3), implying IgG-induced complement activation. By visual assessment, C5b9 staining appears brighter at 2 h compared to 30 min exposure.

To confirm that IgG antibodies are required for A-FT induced neuronal cytotoxicity, we incubated SPMS A-FT with the IgG-degrading enzyme of Streptococcus pyogenes (IdeS), an IgG-specific enzyme that cleaves IgG below the hinge region, yielding F(ab’)2 and Fc fragments [30]. Digestion of MS A-FT with IdeS enzyme completely abolished apoptosis in primary astrocytes, as shown in the MS A-FT dose-response curve (Fig. 3d) and time course (Fig. 3e). To further demonstrate the requirement of IgG antibodies in neuronal apoptosis, we carried out IgG depletion experiments followed by apoptosis assays. IgG1-depleted MS A-FT lost its cell-killing capacity and nearly reached background levels as seen with HC A-FT (Fig. 3f). IgG3 depletion also showed a reduction of neurotoxicity (Fig. 3f). Additionally, direct incubation of anti-IgG1 and anti-IgG3 antibodies with MS A-FT reduced neuron apoptosis compared to controls (Fig. 3g). However, the current anti-B cell therapeutic anti-CD20 antibodies did not reduce cytotoxicity (Fig. 3g). Furthermore, incubation with anti-human IgG (Fc) and anti-human IgG (H + L) antibodies showed a reduction of apoptosis (Fig. 3h). In contrast, the current MS disease-modifying drug Mitoxantrone (MX) did not prevent MS A-FT induced cell death (Fig. 3i).

### Significantly higher levels of neuronal apoptosis were induced by MS plasma IgG antibodies in the A-FT

**Fig. 4:**
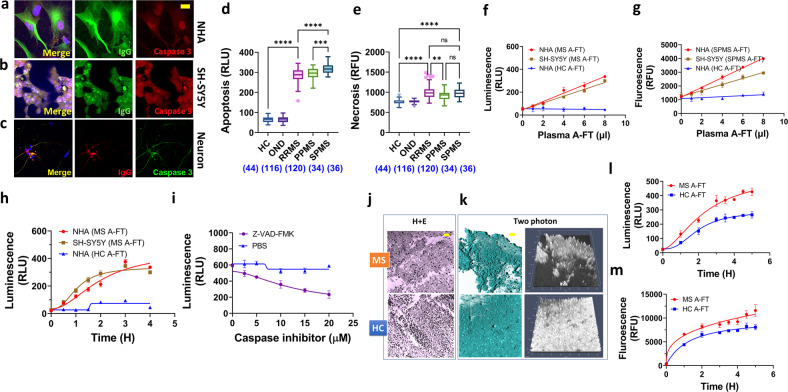
MS plasma IgG in the A-FT induces significantly higher levels of cytotoxicity in neuronal cells. **a**–**c** Confocal images showing co-immunostaining of IgG and apoptosis marker caspase-3 in astrocytes (NHA) (**a**), neuroblastoma (**b**), and human neurons (**c**). IgG antibodies are present on the cell surface and cytoplasm while caspase-3 is shown in both the nucleus and cytoplasm. Bar, 10 µm (**a**, **b**), 30 µm (**c**). **d, e** Plasma IgG in the A-FT of all three MS subtypes (RRMS, PPMS, SPMS) induces about 6 times more apoptosis (**d**) compared to both OND and HC A-FT and significantly higher levels of necrosis in SH-SY5Y cells (**e**). Sample numbers were shown in parenthesis under each category (**p < 0.01; ***p < 0.001; ****p < 0.0001). One-way ANOVA. **f**, **g** Dose-dependent apoptosis (**f**) and necrosis (**g**) is induced by MS A-FT in NHA and SH-SY5Y cells. **h** In SH-SY5Y and NHA cells, MS A-FT caused time-dependent apoptosis, while HC A-FT did not cause significant toxicity. **i** The pan-caspase inhibitor Z-VAD-FMK prevents apoptosis promoted by MS A-FT in a dose-dependent manner. **j** MS plasma IgG in the A-FT induces cell death and tissue damage in newborn mouse brain slices as demonstrated by H + E staining (3 h incubation). Images show that MS A-FT-treated brain tissues (top panel) are damaged/lost, and the nuclei are swelling, resulting in significantly less cell density compared to HC A-FT-treated tissues. **k** Two-photon microscopy demonstrated the acute brain tissue damage caused by MS A-FT, reflected by severe structural impairments. The 3D surface reconstruction and 2.5 D mode intensity plot are shown. **l**, **m** The brain tissue damage and cytotoxicity induced by MS A-FT are quantified by luminescence (apoptosis, **l**) and fluorescence (necrosis, **m**) in a time-dependent manner.

To solidify that neuronal apoptosis was caused by IgG antibodies in the MS A-FT, we carried out immunocytochemistry for the detection of co-deposition of IgG and the apoptosis marker - the activated caspase-3. IgG and caspase-3 were detected in astrocytes, neuroblastoma, and primary neurons (Fig. 4a–c), suggesting that, indeed, apoptotic cell death was induced by IgG antibodies. We provided control images (Supplementary Fig. 4) of cells treated with HC A-FT showing minimum caspase-3 staining in all cell types.

To validate that the cytotoxic IgG in MS is specific, we evaluated MS plasma A-FT for cytotoxicity in primary human astrocytes and SH-SY5Y cells in a large number of samples [MS = 190; other neurological disorders (OND) = 116; HC = 44] from two cohorts. Because similar cytotoxic effects were observed in both neuroblastoma cells and primary neurons, as shown in Fig. 2c, f, g, therefore, we chose SH-SY5Y cells as a surrogate for neurons. In the University of Colorado (CU) discovery cohort, we analyzed 100 MS [68 RRMS, 6 primary progressive MS (PPMS), 26 SPMS], and 140 controls (116 OND and 24 HC). In the confirmatory cohort samples from the Advanced Cure Project (ACP), we included 90 MS (52 RRMS, 28 PPMS, 10 SPMS) and 20 HC. We showed the combined data from the two cohorts that MS A-FT induced about 6 times more apoptotic cells compared to all controls in SH-SY5Y neurons (*p* < 0.0001, Fig. 4d). Similar cytotoxic effects by MS A-FT were observed in primary human astrocytes (data not shown). Furthermore, SPMS A-FT generated significantly higher levels of apoptosis compared to RRMS or PPMS (*p* = 0.0004), and no difference between RRMS and PPMS A-FT was detected (Fig. 4d). These data support that IgG-induced neuronal apoptosis is MS specific, as we failed to see such effects in plasma A-FT from OND/other inflammatory diseases including Parkinson’s, hypertension, headache, Alzheimer’s, traumatic brain injury (Day 30 post impact), lymphoma, viral meningitis, Behcet’s disease, Cryptococcal meningitis, subacute sclerosing panencephalitis, neurosyphilis, sarcoid, VZV myelopathy, and brain tumors (see list of OND in Supplementary Table 1).

The RealTime-Glo™ Annexin V Apoptosis and Necrosis kits include a DNA-binding dye to measure necrosis. Figure 4e shows the highly significant levels of necrosis in neurons caused by MS A-FT (*p* < 0.0001). Interestingly, RRMS A-FT treated cells showed higher levels of necrosis compared to PPMS (Fig. 4e). In addition, we demonstrated that MS A-FT induced apoptosis in NHA and SH-SY5Y (measured by luminescence) was accompanied by necrosis (measured by fluorescence) in a dose- and time-dependent manner (Fig. 4f–h). Furthermore, we showed that neuronal apoptosis can be inhibited by the pan-caspase inhibitor Z-VAD-FMK, which corroborates the cell death mechanism is likely initiated by apoptosis (Fig. 4i).

We treated newborn mouse brain slices with MS A-FT. Compared to controls, MS A-FT generated severe neuronal cell injuries in the brain tissues as exhibited by a gross lower cell number and obvious structural damage. Figure 4j–k are representative images of H + E staining and two-photon microscopy. The brain tissue damage and cytotoxicity were quantified by luminescence (apoptosis, Fig. 4l) and fluorescence (necrosis, Fig. 4m) in a time-dependent manner.

### MS plasma A-FT contained the most potent IgG antibodies for neuronal cytotoxicity

**Fig. 5:**
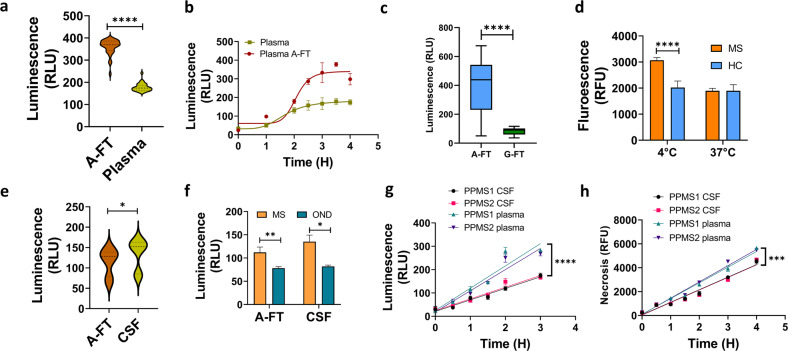
Significantly higher levels of neuronal cytotoxicity were induced by MS plasma A-FT compared to paired neat plasma, Protein G flow-through, and paired CSF. **a** IgG antibodies in MS plasma A-FT induced significantly higher levels of apoptosis in neurons compared to that of paired plasma (****p < 0.0001, *n* = 32), Mann-Whitney U test. **b** Time course shows higher levels of apoptotic cell death induced by MS A-FT compared to paired plasma. **c** Significantly higher levels of apoptosis were caused by MS plasma A-FT, but not by paired Protein G flow-through (G-FT) (*p* < 0.0001, *n* = 29). Mann-Whitney U test. **d** MS A-FT prepared at 4 °C generated neuronal cytotoxicity but not the A-FT collected at 37 °C, indicating the temperature-sensitive nature (MS A-FT vs HC A-FT *p* < 0.0001, *n* = 8). **e** The protein A flow-through of MS CSF induced lower levels of apoptosis compared to paired CSF (*p* = 0.0163, *n* = 7). **f** Both MS CSF and CSF A-FT promoted higher levels of apoptosis compared to OND. CSF: *p* = 0.0218, MS = 7, OND = 8; CSF A-FT: *p* = 0.0412, MS = 8, OND = 5. **g**, **h** MS plasma A-FT induced higher levels of apoptosis (*p* < 0.0001) (**g**) and necrosis (*p* = 0.0002) (**h**) compared to paired CSF A-FT in a time-dependent manner.

We compared the induction of apoptosis by MS plasma A-FT and paired original plasma. We found that MS plasma A-FT induced two times higher levels of cell death in neurons compared to paired plasma (Fig. 5a, *p* < 0.0001). Figure 5b shows time-dependent apoptosis. Further, we showed that apoptosis induction was 5 times higher by MS plasma A-FT than paired plasma Protein G flow-through (G-FT) (Fig. 5c, *p* < 0.0001). Interestingly, we discovered that MS A-FT prepared at 4 °C, but not at 37 °C, contained cytotoxic IgG, indicating the temperature-sensitive feature of the IgG aggregates (Fig. 5d, *p* < 0.0001).

We evaluated the cytotoxicity by MS CSF A-FT in neuroblastoma cells. To our surprise, MS CSF A-FT was not as toxic as original CSF (Fig. 5e, *p* = 0.0163), although both MS CSF and CSF A-FT induced significantly higher levels of apoptosis than that from OND (*p* = 0.0002, Fig. 5f). Furthermore, contrary to the common belief that MS CSF is more toxic, we showed that PPMS plasma A-FT induced higher levels of apoptosis (Fig. 5g) and necrosis (Fig. 5h) compared to paired CSF A-FT in a time-dependent manner.

### The cytotoxic IgG antibodies in MS plasma A-FT are contributed by IgG aggregates (>100 nm) which can be disrupted by 8 M urea and low pH buffer

**Fig. 6:**
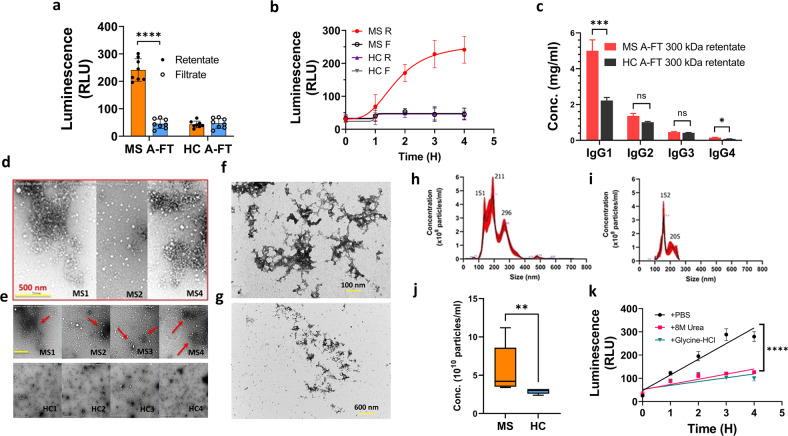
MS plasma IgG antibodies form IgG aggregates which were retained in the retentates after 300 kDa filtration. **a** Only the 300 kDa retentates but not the filtrates of MS A-FT drove apoptosis in neurons (*p* < 0.0001, *n* = 8 each). **b**. Time-dependent apoptosis is shown by 300 kDa retentates of MS A-FT (R Retentate; F Filtrate). **c** Human IgG subclass ELISA shows significantly higher levels of IgG1 in MS 300 kDa retentates compared to HC (MANOVA *p* < 0.0001, *n* = 8 each). **d** Close-up views of TEM images demonstrating the IgG aggregates in the 300 kDa retentates from three individual MS A-FT. **e** TEM images showed that only MS samples (top panel) contained the IgG aggregates (red arrows); in HC A-FT 300 kDa retentates the IgG aggregates were essentially absent (lower panel). Scale bar: 500 nm. Uranyl acetate was used for staining. **f**, **g** TEM images of IgG aggregates in MS A-FT stained with uranyl formate at high (**f**) and low (**g**) magnification. Scale bar: 100 nm (**f**), and 600 nm (**g**). **h**–**j** Nanoparticle Tracking Analysis (NTA) demonstrates that MS A-FT retentates (**h**) contained large particles (>100 nm) compared to HC A-FT (**i**). **j** Summary of NTA data showing that MS A-FT 300 kDa retentates contained twice the amounts of particles (> 100 nm) compared to HC (**p < 0.01, *n* = 4 each). **k** Disruption of the IgG aggregates abolished MS A-FT-induced apoptosis. Incubation with 8 M urea or 0.2 M Glycine-HCl, pH 2.8 abolished MS A-FT-induced neuronal apoptosis in a time-dependent manner. Pooled MS A-FT (*n* = 8) were incubated with 8 M urea or 0.2 M Glycine-HCl (1:3) at 37 °C for 1 h, followed by the collection of the 300 kDa retentates after 300 kDa and apoptotic assay.

To further characterize the IgG antibodies in the A-FT and prove that the IgG antibodies form aggregates, we collected both retentates (R) and filtrates (F) after filtration of MS A-FT using 300 kDa cut-off centrifugal membranes. Only the 300 kDa retentates, but not the filtrates, generated apoptosis (Fig. 6a. *p* < 0.0001). Figure 6b shows time-dependent apoptosis caused by the 300 kDa retentates of MS A-FT. We further carried out ELISA and confirmed the presence of elevated levels of IgG1 in MS A-FT 300 kDa retentates (MANOVA, *p* < 0.0001). These data support that the cytotoxic IgG in MS A-FT may be contributed by IgG aggregates (>300 kDa) which is consistent with findings by Diebolder and colleagues [31]. In addition, we examined the structures of the IgG aggregates from the 300 kDa retentates using transmission electron microscopy (TEM). We demonstrated that MS samples contained large IgG aggregates about 100 nm in size (Fig. 6d–g), exhibiting clusters of aggregates as shown in high and low-power magnification images. In contrast, there were few IgG aggregates in HC 300 kDa retentates (Fig. 6e). Our data are consistent with a previous study showing similar sizes of IgG aggregates [32]. We further evaluated the IgG aggregates using nanoparticle tracking analysis (NTA) and confirmed that MS A-FT 300 kDa retentates contained twice the amounts of particles (>100 nm) compared to controls (Fig. 6h–j. *p* < 0.01, n = 4 each). Additional data revealed that disruption of the IgG aggregates with 8 M urea or 0.2 M Glycine-HCl, pH 2.8, abolished MS A-FT-induced neuronal apoptosis (Fig. 6k).

## Discussion

Axonal loss is a key pathological feature of MS demyelinating lesions. Neuron-specific activation of necroptosis was reported recently in MS cortical grey matter [16]. The IgG antibody-induced complement activation and neuronal apoptosis may be a novel mechanism for axonal loss and neuronal degeneration in MS. Neuronal degeneration and white matter demyelination can be independent events in the disease [33]. Progressive MS CSF has been shown to induce demyelination and neuronal apoptosis [34, 35]. In addition, MS plasma antibodies were shown to target brain micro-vessels, disrupt the blood-brain barrier, and correlate with brain MRI measures of disease severity [36–38]. Our data are consistent with these studies and support the pathological roles of IgG antibodies in MS [25].

We demonstrated MS plasma IgG-induced complement activation by C1q depletion assay, immunocytochemistry, and blocking of C1q and C3b in treated neurons. The detection of complement components supports complement-mediated neuronal cytotoxicity. The involvement of MS IgG antibodies in neuronal apoptosis was supported by data from IgG digestion, IgG antibody depletion, and IgG blocking experiments. In addition, we demonstrated the presence of IgG aggregates in MS A-FT by filtration experiments, transmission electron microscopy, and nanoparticle tracking analysis. Furthermore, the IgG aggregate disruption data support the role of IgG aggregates in causing neuronal death. Although Lisak and colleagues reported that MS blood B cell culture supernatants were cytotoxic to human neurons [24], they failed to detect IgG antibodies.

Activated complement is a component of central inflammation in MS, both in the early and later progressive phases [39, 40]. The universal presence of IgG antibodies and C1q staining in MS plaques suggests a dominant role for IgG and the classical complement pathway [13, 14]. The detection of antibody-antigen immunocomplexes in foamy macrophages in the active lesion areas in MS [41], and findings that demyelination by autoantibodies requires complement activation without Fc receptor activation [42], support our data of IgG-mediated complement activation in MS. A recent study demonstrating a strong association between neurodegeneration and local complement activation in the thalamus of progressive MS [43] further supports our results.

C1q binds to monomeric IgG with very low affinity but binds to IgG hexamers with high avidity promoting efficient complement activation [31], leading to tissue damage and dynamic systemic activation of complement [44, 45]. MS patients with a greater number of chronic active lesions harbor more risk variants in early complement genes, including C1q and C3 [18]. Our findings are consistent with these studies.

Protein A (from *Staphylococcus aureus*) binds with high affinity to the Fc region of immunoglobulins [46] and has been extensively used for the purification of IgGs [47]. It was shown recently that Protein A inhibits complement activation by interfering with IgG hexamer formation [48]. Our A-FT IgG aggregate data suggest that the MS IgG1 and IgG3 antibodies may possess unique Fc regions, and form aggregates or IgG complexes that prevent binding to Protein A. In addition, the large IgG aggregates in MS may be formed by non-covalent interactions in the Fc regions which prevent binding to Protein A [31], resulting in the enrichment of IgG aggregates in A-FT and in the 300 kDa retentates. We do not know whether the aggregates of MS plasma IgG depend on specific antigen binding, although MS circulating antibody was shown to bind to myelin and caused demyelination [49]. The oligoclonal bands in MS have been shown to target random intercellular antigens [50] or patient-specific antigens [51]. These findings combined with our proteomics data of higher levels of IgG heavy/light chains and complement components suggest that MS IgG may be antigen-nonspecific. In fact, the immunomodulatory effect of MS IgG aggregates/IgG immune complexes can be either antigen-specific, nonspecific, or a combination of both [52]. Further, it is known that the IgG immune complexes may engage multiple Fc gamma receptors (FcγRs) through low-affinity, high-avidity interactions [53].

Our results are supported by earlier studies [49] that MS plasma with natural complement produced demyelination in newborn rat cerebella. The pathological antibodies in their natural states, possibly forming immune complexes with other antibodies/antigens, are transported across the BBB via Fc receptors and resulting in demyelination [54]. We showed that apoptosis is caused by IgG antibodies in MS A-FT, but not in G-FT, suggesting a unique Fc region of MS IgG and that both IgG1 and IgG3 are critical for cell killing. Furthermore, MS plasma A-FT purified from 37 °C incubation lost cytotoxicity indicating that the IgG aggregates are temperature sensitive and that at the higher temperature, the naturally formed loose aggregates/complexes may be disrupted. This may partially explain why only 30% of the purified plasma IgG in MS produced demyelination [55]. We also found that the purified plasma IgG obtained by negative selection with Melon Gels (Thermo #45206) did not cause neuronal cytotoxicity (data not shown). Indeed, antibodies that efficiently form hexamers upon antigen binding can induce complement-dependent cytotoxicity [56]. MS CSF has shown reduced cell-killing effects compared to paired plasma, even though 125 times more CSF was used compared to the plasma, suggesting that plasma IgG plays a critical role in disease pathology in the CNS [25, 27, 54]. MS plasma IgG concentration (10 mg/ml) is about 200 times higher than paired CSF IgG (about 0.05 mg/ml) [54]. Therefore, plasma IgG may form higher levels of IgG aggregates causing elevated levels of cytotoxic effects.

Insufficient disease inhibition by intrathecal rituximab in progressive MS supports that plasma, not CSF may be the key [57]. Link [58] failed to find any immunochemical difference between CSF and serum-derived IgG in MS patients, and Tourtellotte et al. [59] have shown that serum-derived IgG has the same half-life in CSF as in serum; these findings further support the notion that the CSF IgG may have a systemic origin [54]. We hypothesize that plasma IgG in progressive MS may be able to enter the CNS through the intact blood-brain barrier (BBB) by binding to the neonatal Fc receptor (FcRn). The expression of FcRn was found at the brain microvasculature and choroid plexus epithelium [60]. It has been shown that FcRn transports IgG across cellular barriers between mother and offspring [61]. Similarly, FcRn in the BBB may facilitate the transfer of MS plasma IgG antibodies into CNS. Importantly, a recent study reported that reduced serum levels of IgG were observed in MS patients treated with Rituximab [62]. These data, combined with the report of reduced CSF OCBs after effective B cell therapies, support the pathological role of IgG antibodies in MS [25]. It is possible that in MS there exists a combination of selective transport, a small BBB leakage, and intrathecal synthesis contributing IgG to the CSF IgG [54].

We speculate that IgG antibodies and the antibody-secreting cells, together with CNS B cells, play a critical role in disease pathogenesis. The contradiction between the lower number of CNS cells (about 3.5 million cells in MS CSF) and higher levels of intrathecal IgG (10 mg/day) raises the question as to whether B cells alone in MS CNS can be responsible for the massive amounts of elevated intrathecal IgG and support the pathological role of IgG antibodies in MS [54].

In summary, we discovered that MS plasma IgG antibodies form aggregates that generate complement-dependent apoptosis in neurons and astrocytes. Our findings provide a direct link between IgG antibodies and neuron death which may stipulate strategies for novel therapeutics to prevent neurodegeneration and disease progression. Screening small compound drug libraries may identify inhibitors preventing MS IgG-induced neuronal apoptosis.

## Materials and Methods

### Plasma and cerebrospinal fluid (CSF) samples

With the approval of the University of Colorado Institutional Review Board (COMIRB #00–688, #13-3007), plasma and CSF from MS and control patients with other central nervous systems (CNS) pathologies were collected at the University of Colorado Hospital. Plasmas were collected after centrifugation of blood samples at 2000 × g for 10 min; CSFs were immediately centrifuged at 500 × g for 10 min, and the supernatant was collected. Both CSF and plasma were stored at −80 °C until use. Additional plasma samples (second cohort) including primary progressive MS (PPMS), secondary progressive MS (SPMS), relapse-remitting MS (RRMS), and age and sex-matched healthy controls (HC) were obtained from Accelerated Cure Project (ACP, https://www.acceleratedcure.org/). The plasma samples from brain tumor patients were obtained from the Nervous System Biorepository of the Department of Neurosurgery at the University of Colorado Anschutz Medical Campus (https://medschool.cuanschutz.edu/neurosurgery/research-and-innovation/centers/nervous-system-biorepository). Patient diagnosis, age, and sex are listed in Supplemental Tables 1–3.

### Preparation of Protein A and Protein G flow-through

We chose to use Protein A-coated 96-well plates for collecting the flow-through because of the high-throughput nature. Plasma (2 µl plasma plus 198 µl of PBS, Gibco #10010-023) or CSF (150 µl plus 50 µl of PBS) were added to wells of Protein A-coated 96-well plates (Thermo #15130) and incubated at 4 °C for 2–5 h. The unbound solution/supernatant named #1 Protein A flow-through (A-FT) was transferred to a second well and incubated overnight at 4 °C. The flow-through collected was named #2 A-FT. Similar procedures were carried out for collecting #3 and the final flow-through except the incubation time was 2 h each. The final A-FT was collected, aliquoted, and stored at −80 °C. The collections of Protein G flow-through (G-FT) were the same except for using Protein G-coated 96-well plates (Thermo #15131).

### IgG subclass ELISA

The human IgG subclass ELISA kit (Invitrogen #991000) was used to determine the concentrations of IgG subclasses (IgG1–4) in plasma and A-FT samples. The A-FT and plasma samples, pre-diluted at 1:100 in PBS, were further diluted at 1:30 using the dilution buffer provided by the kits (total dilution of 1:3000). Fifty µl of the corresponding antibody (MAB anti-hIgG1, 2, 3, 4) were added to each well, followed by the addition of 50 µl of diluted samples or standards. The plate was incubated for 1 h at room temperature at 25 rpm. After washing 6 times, the plate was incubated with HRP-anti-human IgG (1:50 dilution) followed by TMB substrate incubation. The color intensity was determined by a microplate reader (BioTek Synergy H2 with Gen5 1.11 software).

### Mass spectrometry proteomic analysis of Protein A flow-through

Note: in this section, “MS” will mean “mass spectrometry”.

#### Sample digestion

The samples were digested according to the FASP (Filter-Aided Sample Preparation) protocol using a 10 kDa molecular weight cutoff filter. In brief, the samples were mixed in the filter unit with 200 µl of 8 M urea, 0.1 M ammonium bicarbonate (AB) pH 8.0, and centrifuged at 14,000 *g* for 15 min. The proteins were reduced with 10 mM DTT for 30 min at room temperature (RT), centrifuged, and alkylated with 55 mM iodoacetamide for 30 min at RT in the dark. Following centrifugation, samples were washed 3 times with urea solution, and 3 times with 50 mM AB. Protein digestion was carried out with sequencing grade modified Trypsin (Promega) at 1/50 protease/protein (wt/wt) at 37 °C overnight. Peptides were recovered from the filter using 50 mM AB. Samples were dried in Speed-Vac and desalted and concentrated on Thermo Scientific Pierce C18 Tip.

#### Mass spectrometry analysis

Samples were analyzed on an Orbitrap Fusion mass spectrometer (Thermo Fisher Scientific) coupled to an Easy-nLC 1200 system (Thermo Fisher Scientific) through a nanoelectrospray ion source. Peptides were separated on a self-made C18 analytical column (100 µm internal diameter, ×20 cm length) packed with 2.7 µm Cortecs particles. After equilibration with 3 µl 5% acetonitrile 0.1% formic acid, the peptides were separated by a 120 min linear gradient from 6% to 38% acetonitrile with 0.1% formic acid at 400 nL/min. LC mobile phase solvents and sample dilutions used 0.1% formic acid in water (Buffer A) and 0.1% formic acid in 80% acetonitrile (Buffer B) (Optima™ LC/MS, Fisher Scientific). Data acquisition was performed using the instrument supplied Xcalibur™ (version 4.1) software. Survey scans covering the mass range of 350–1800 were performed in the Orbitrap by scanning from *m/z* 300–1800 with a resolution of 120,000 (at *m/z* 200), an S-Lens RF Level of 30%, a maximum injection time of 50 milliseconds, and an automatic gain control (AGC) target value of 4e5. For MS2 scan triggering, monoisotopic precursor selection was enabled, charge state filtering was limited to 2–7, an intensity threshold of 2e4 was employed, and dynamic exclusion of previously selected masses was enabled for 45 s with a tolerance of 10 ppm. MS2 scans were acquired in the Orbitrap mode with a maximum injection time of 35 milliseconds, quadrupole isolation, an isolation window of 1.6 m/z, HCD collision energy of 30%, and an AGC target value of 5e4.

MS/MS spectra were extracted from raw data files and converted into mgf files using Proteome Discoverer Software (ver. 2.1.0.62). These mgf files were then independently searched against the human database using an in-house Mascot server (Version 2.6, Matrix Science). Mass tolerances were +/− 10 ppm for MS peaks, and +/− 25 ppm for MS/MS fragment ions. Trypsin specificity was used allowing for 1 missed cleavage. Met oxidation, protein N-terminal acetylation, and peptide N-terminal pyroglutamic acid formation were allowed as variable modifications. The Scaffold (version 4.8, Proteome Software) was used to validate MS/MS-based peptide and protein identification. Peptide identifications were accepted if they could be established at greater than 95.0% probability as specified by the Peptide Prophet algorithm. Protein identifications were accepted if they could be established at greater than 99.0% probability and contained at least two identified unique peptides.

The partial least squares-discriminant analysis (PLS-DA) and heatmaps were performed using the MetaboAnalyst 5.0 online platform with sum and range scaling normalizations. Proteomic data were further analyzed using QIAGEN Ingenuity Pathway Analysis (IPA; QIAGEN Inc. https://digitalinsights.qiagen.com/IPA (accessed 27 October 2022). Venn diagrams were generated using Venny 2.1 (https://bioinfogp.cnb.csic.es/tools/venny/index.html) (accessed 27 October 2022).

### Neuronal cells and mouse brain tissues

The 96-well tissue culture plates (BD Falcon #353219) and 12 mm glass coverslips (Corning #354087) in the 24-well plate (BD Falcon #353226) were used for all cell cultures.

#### Neuroblastoma cell line SH-SY5Y

The SH-SY5Y neuroblastoma cells were obtained from ATCC (#CRL-2266). Cells were cultured in DMEM with 10% FBS and 1X Penicillin-Streptomycin on 0.1% gelatin-coated plates, passaged with 0.05% Trypsin and 0.02% EDTA solution. Cells at passage number 3–10 were used for cytotoxicity assays. For MS A-FT treatments, cells were plated at 20,000/cm^2^ in 96-well plates. For immunocytochemistry, cells were plated at 20,000/cm^2^ in Poly-D-Lysine/Laminin-coated 12 mm glass coverslips in 24-well tissue culture plates. The cells were treated on days 3–5 at 30–50% of confluency.

#### Human primary neurons

Human primary neurons were obtained from ScienCell (#1520). The frozen cells (passage 0, 1 × 10^6^ cells) were thawed at 37 °C and plated on Poly-D-Lysine/Laminin-coated 12 mm coverslips in a 24-well plate. The neurons were also plated on Poly-L-Lysine (2 µg/cm^2^) coated 96-well plates at a density of 10,000 cells/cm^2^. The culture medium consisted of 98% Neuronal Medium (ScienCell #1521), 1% Neuronal Growth Supplement (ScienCell #1562), and 1X Penicillin/Streptomycin. The medium was changed every 2 to 3 days. The neurons were treated on Day 5 after plating.

#### Human primary astrocytes

Human primary astrocytes were obtained from ScienCell (#1800). The frozen cells (passage 1, 1 × 10^6^ cells) were thawed and plated on Poly-L-Lysine (2 µg/cm^2^) coated 10-cm tissue culture plate. The culture medium consisted of 96% Basal Medium (ScienCell #1801), 2% FBS (ScienCell #0010), 1% Astrocytes Growth Supplement (#1852), and 1X Penicillin/Streptomycin. The cells were passaged with 0.05% Trypsin/0.02% EDTA and plated on Poly-L-Lysine (2 µg/cm^2^) coated 96-well plates and Poly-D-Lysine/Laminin coated 12 mm coverslips in 24-well plate, at a density of 5000 cells/cm^2^. The medium was changed every 2 to 3 days. The astrocytes were treated on days 3–5 after plating at 30–50% confluency.

#### Newborn mouse brain slices

The animal protocols were approved by the University of Colorado Institutional Animal Care and Use Committee (IACUC) and conform to the National Institutes of Health guidelines for the care and use of animals in research. P1 C57BL/6 mice were purchased from Charles River Laboratories (Wilmington, MA). Animals were housed with a standard 12 h light-dark cycle with free access to food and water. Newborn mice (P1) were anesthetized by hypothermia and euthanized by decapitation. Cerebral tissue was dissected and kept in cold PBS. Brain tissues were cut into thin slices at about 1 mm in thickness followed by cutting into 2 mm rounds with a tissue biopsy puncher (Zivic Instruments Inc, PUN2000). The tissue slices were maintained in a 4-chamber glass-bottom 35 mm dish (Cellvis #D35C4-20-1-N) in a human neural basal A medium supplemented with 10 µg/ml bFGF and 10 µg /ml EGF.

### Evaluation of cytotoxicity of cells treated with MS A-FT

#### MS A-FT treatments

SH-SY5Y cells, human primary neurons, and human primary astrocytes, at 30–50% of confluency were used for treatment. The following components were mixed in a 96-well U-bottom sterile plate (Greiner Bio-One #6018-P113): 1) samples (plasma A-FT, plasma G-FT, plasma, CSF A-FT or un-diluted CSF); 2) 5% Normal Human Serum (NHS) as a source of complement (Complement Technology Inc, #NHS); 3) 1x assay reagents (Apoptosis/Necrosis Assay, Live/Dead Assay, or Propidium Iodide); 4) culture medium to a final volume of 100 µl for 96-well plates and 200 µl for 24-well coverslips. After removing the spent media, cells were washed once with a fresh warm medium, followed by incubation with the treatment mixtures. The cytotoxicity was measured every 30–60 min for 4–24 h. All readings were carried out using a Biotek Synergy H4 hybrid reader (software version Gen5 3.09).

#### Propidium iodide and LIVE/DEAD viability/cytotoxicity assays

We used Propidium iodide (5 µg/ml, Thermo # P3566) (Excitation/Emission: 535/617 nm) for the initial evaluation of damaged/dead cells. LIVE/DEAD™ Viability/Cytotoxicity Kit was used for quantifying cell viability (Invitrogen #L3224). Calcein AM (1 µM) was used for labeling live cells and the Ethidium Homodimer-1 (2 µM) for labeling dead cells. The live and dead cells were quantified by green fluorescent (Excitation/Emission: 485/528 nm) and red fluorescent (Excitation/Emission: 535/617 nm), respectively.

#### Apoptosis/Necrosis assays

The RealTime-Glo Annexin V Apoptosis and Necrosis Assay kit (Promega #JA1011) was used for evaluating apoptotic and necrotic cell death. The five components (stock concentration at 1000×) were diluted to 1x in the culture medium and added to the treatment mixtures. Apoptosis was measured by luminescence and necrosis by green fluorescence (Excitation/Emission: 485/528 nm).

#### Confocal microscopy

After A-FT treatments, cells were fixed with cold 4% paraformaldehyde for 30 min followed by 3X PBS washing and stored at 4 °C. Cells were treated with 5% Normal Horse Serum (blocking buffer)/0.1% Triton X-100 for 30 min at room temperature, followed by incubation with primary antibodies diluted in blocking buffer overnight at 4 °C. After washing, cells were incubated with respective secondary antibodies for 2 h at room temperature in the dark. After the final washes with PBS, cells on coverslips were mounted in an anti-fade medium with DAPI or Hoechst 33342 (1 µM). Supplemental Table 4 lists all antibodies used.

The stained cells were examined with a Zeiss LSM 788 confocal microscope. Two to five random fields were chosen for imaging with optics at 63×. The laser power was set at the same intensity for experimental and control wells.

#### Two-photon microscopy

After overnight incubation, the mouse brain slices in triplicates were treated with 10 µl of HC A-FT and MS AFT with 5% NHS for 1 or 3 h. The tissues were washed 3 times with a fresh culture medium. The treated and untreated live brain tissues were examined with two-photon confocal microscopy (Zeiss LSM 780) with a tunable (690 nm–1040 nm) infrared pulsed laser (MaiTai, SpectraPhysics) to evaluate tissue damage. The setup is equipped with an environmental chamber (37 °C, 5% CO_2_, and humidity control) for live tissue imaging. A C-Apochromat 40x/1.20 W Korr FCS M27 objective was used for imaging brain tissues excited with the two-photon laser tuned at 800 nm. Two-photon microscopy max intensity projection was obtained from Z-stacks to visualize structural images in deep tissues. 3D intensity projection (XY image size x: 1024, y: 1024, Z: 33, 8-bit) was reconstructed from Z-stacks (sample x: 353.90 µm, y:353.90 µm, z: 10.65 µm, 33 slides) in scan mode with a pixel dwell time of 1.58 ms, open pinhole, spectral emission filter set to detect the whole visible range. The 2D maximum intensity projection image size was 1024 × 1024 resolution.

#### H&E staining

For histology, mouse cerebral tissue slices were treated with MS A-FT or HC A-FT for 3.5 h and the tissues were fixed with 4% paraformaldehyde for 30 min at RT. The tissues were paraffin-embedded and cut at 4 µm sections for H&E staining.

### Characterization of IgG and IgG aggregates in Protein A flow-through

We used pooled SPMS A-FT and HC A-FT (*n* = 10 each) for characterization experiments.

#### Inhibition of complement components

C1q-depleted human serum (Complement Technology #A300_C1q-Dep) was used to replace normal human serum in cell treatment. To test if C1q and C3b complements are required for MS IgG-mediated cytotoxicity, the following antibodies in various doses were pre-mixed with MS-AFT before exposing to cells: rabbit anti-human C1q antibody (DAKO, A0136), rabbit anti-human C3b (Mybiosource MBS2559642).

#### Digestion of IgG antibodies with IdeS

Protein A flow-through was incubated with an IgG-specific enzyme IdeS (FabRICATOR, Genovis #A0-FR1-008) to produce a homogenous pool of F(ab’)2 and Fc/2 fragments. Five units of IdeS were used to digest 20 µl of A-FT at 37 °C for 30 min. For controls, an equal amount of PBS was used to replace the enzyme in each digestion. The digested A-FT were kept on ice before being used for cell treatments.

#### Depletion of IgG1 and IgG3 in MS A-FT

Pooled SPMS A-FT (20 µl) were incubated with 15 µl of biotinylated mouse anti-human IgG1 antibody (Sigma #B6775, 4 mg/ml), or anti-human IgG3 antibody (Southern Biotech #921008, 0.5 mg/ml) in a final 100 µl volume of PBS, and incubated at RT for 2 h, followed by overnight incubation at 4 °C. M-280 Streptavidin-conjugated Dynabeads (Invitrogen #11205D) were used to capture the biotinylated antibodies (depletion). The unbound solutions were collected as IgG1-, and IgG3-depleted A-FT.

#### Inhibition of IgG antibody-mediated apoptosis

To test if there is a dose-dependent cytotoxicity-blocking effect with anti-human IgG antibodies, cells were treated with 5 µl of MS or HC A-FT, 5% NHS, plus various amounts of anti-human IgG antibodies. The following blocking antibodies were used: mouse anti-human IgG1 (Sigma #I2513), mouse anti-human IgG3 (Sigma #SAB4200759), goat anti-human IgG (H + L) (Vector Lab, #AI-3000), goat anti-human IgG-Fc (Rockland # 609-1103), and mouse anti-CD20 (R&D system #MAB4225). In addition, we tested MS drug Mitoxantrone (Cayman Chemical #14842) and pan-caspase inhibitor Z-VAD-FMK (Promega #G7231).

#### Comparison of cytotoxicity by plasma and paired CSF A-FT

Various amounts of plasma A-FT (0, 2, 4, 6 µl) and CSF A-FT (0, 2, 4, 10 µl) were added to SH-SY5Y cells as described above. Cytotoxicity was measured by luminescence and green fluorescence for 0–4 h after incubation.

#### Collection of retentates and filtrates after 300 kDa filtration of plasma A-FT

We used a 300 kDa centrifugal device Nanosep 300 K OMEGA (Pall Life Science, #OD300C34) for the collection of the IgG aggregates in the retentates. MS and HC A-FT diluted in Ca^2+^-Mg^2+^-free PBS were centrifuged for 10 min at 6000 x g, and both the filtrates and the retentates (in PBS) were collected.

#### Negative staining of IgG aggregates and visualization via transmission electron microscopy (TEM)

The 300 kDa A-FT retentates were used for TEM imaging.

*1) Staining with uranyl acetate*. Samples were prepared on continuous carbon films supported on 200 mesh copper grids (Ted Pella). A 2.5 μl drop of MS or control retentate was applied to a freshly glow-discharged grid for 20 s and blotted with filter paper. The grid was then washed one time by dipping in a droplet of water for 15 s followed by staining on a droplet of 2% (w/v) uranyl acetate for 1 min. Grids were blotted after each incubation and air-dried after blotting excess stain solution. Stained grids were imaged using a Thermo-Fisher Talos L120C transmission electron microscope outfitted with a LaB6 filament and operating at an acceleration voltage of 120 kV. Automated data collection was carried out using Leginon, with images recorded using a Ceta CMOS detector at a magnification of 36,000X (3.98 Å/pixel).

*2) Staining with uranyl formate*. Samples were diluted at 1:20 in PBS. The copper mesh grid coated with formvar and carbon (Electron Microscopy Sciences) was used. Samples (3 µl) were absorbed into the grid for 1 min and then gently blotted off with a piece of Whatman filter paper. The grids were rinsed with transfers between six drops of MilliQ water, 1 min each. Finally, the grids were stained with 10 ml of 2% uranyl formate solution for 3 min, followed by three washes with MilliQ water. After blotting, the grids were allowed to dry. Samples were imaged on an FEI Tecnai G2 Biotwin TEM (ThermoFisher) at 80 kV with an AMT side-mount digital camera (AMT Imaging).

#### Nanoparticle tracking analysis

MS IgG aggregates were determined by Nanoparticle Tracking Analysis (NTA) with a Nanosight device (NS300, Software Version: NTA 3.2 Dev Build 3.2.16; Malvern P analytical). The IgG aggregates were captured using the following settings: camera type: CMOS, level 11; green laser; slide setting and gain: 600, 300; shutter: 15 milli-seconds; histogram lower limit: 0; upper limit: 8235; frame rate: 24.9825 fps; syringe pump speed: 25 arbitrary units; detection threshold: 7; max jump mode: auto; max jump distance: 15.0482; blur and minimum track length: auto; first frame: 0; total frames analyzed: 749; temp: 21.099–22.015 °C; viscosity: 1.05 cP. The experiments were repeated at least 3 times.

#### Disruption of IgG aggregates

Pooled MS A-FT was incubated with 8 M urea (Sigma#51457), or 0.2 M Glycine/HCI, pH 2.8, or PBS (1:3 sample to buffer ratio v/v) at 37 °C for one hour, followed by addition of PBS and filtration through 300 kDa Nanosep spin column (described above). The retentates were collected for apoptosis assays and IgG subclass ELISA as described above.

### Statistical analysis

All experiments were repeated at least twice. Statistical analyses were performed using GraphPad Prism (version 8.3). A one-way analysis of variance (ANOVA) test with a post hoc Tukey’s multiple comparisons test was performed to determine the significance of the results. Mann–Whitney U test was used for unpaired samples. A Tukey-adjusted multivariate ANOVA (MANOVA) was used to simultaneously compare each of the four IgG subclasses to the control group. *P*-values < 0.05 were considered statistically significant and all tests were two-sided.

## Supplementary information

Supplemental Tables and Figure

CDD AJ-Checklist

## Data Availability

All data are available for review and materials are available upon reasonable request.
