# Supplementary material for: Multiple sclerosis plasma IgG aggregates induce complement-dependent neuronal apoptosis
Source: Cell Death Dis. 2023 Apr 8;14(4):254. doi: 10.1038/s41419-023-05783-3 (PMC10082781; doi:10.1038/s41419-023-05783-3)
Supplement: Supplementary file 1 — Supplemental Tables and Figure [file 41419_2023_5783_MOESM1_ESM.docx]

**Multiple sclerosis plasma IgG aggregates induce complement-dependent neuronal apoptosis**

Wenbo Zhou^1#^, Michael Graner^1#^, Petr Paucek^1^, Cheryl Beseler^2^, Matthew Boisen^3^, Andrew Bubak^4^, Francisco Asturias^5^, Woro George^1^, Arin Graner^1^, David Ormond^1^, Timothy Vollmer^4^, Enrique Alvarez^4^, Xiaoli Yu^1^*

**Supplemental Data**

Supplemental Table 1: CU/IIL (Cohort 1) MS and control subject demographics

Supplemental Table 2: ACP (Cohort 2) MS and Control subject demographics

Supplemental Table 3: CSF sample patient demographics

Supplemental Table 4: Antibodies and Reagents used in the study

Supplemental Figure 1: Top three networks obtained from proteomic data of A-FT

Supplemental Figure 2: Complement-based networks obtained from IPA

Supplemental Figure 3: Confocal images of cells treated with healthy control (HC) A-FT and time course of MS A-FT treated cells

Supplemental Figure 4: Confocal images for IgG and caspase 3 in three types of cells treated with HC A-FT

**Supplemental Table 1****. CU/IIL (Cohort 1) MS and control subject demographics.**

| **Variable** | **Primary progressive MS (PPMS)**  **PPMS** | **Secondary progressive MS (SPMS)** | **Relapsing remitting MS (RRMS)**  **(RRMS)** | **Healthy Control (HC)** | **Other Neurological Disorders (OND)*** |
| --- | --- | --- | --- | --- | --- |
| **Subject (n)** | 6 | 26 | 68 | 24 | 116 |
| **Male (n)** | 4 | 9 | 15 | 9 | 58 |
| **Female (n)** | 2 | 17 | 53 | 15 | 58 |
| **Age (years, mean± SD)** | 49.0 ± 15.5 | 55.7 ± 8.4 | 42.5 ± 11.7 | 44.7 ± 11.5 | 53.1 ± 15.4 |

* OND, Other neurological disorders including Parkinson’s, hypertension, headache, Alzheimer’s, traumatic brain injury (Day 30), lymphoma, viral meningitis, Behcet's disease, Cryptococcal meningitis, subacute sclerosing panencephalitis, neurosyphilis, sarcoid, VZV myelopathy, and brain tumors.

**Supplemental Table 2****. ACP (Cohort 2) MS and control subject demographics.**

| Variable | Primary progressive MS (PPMS) | Secondary progressive MS (SPMS) | Relapsing remitting MS (RRMS) | Healthy Control (HC) |
| --- | --- | --- | --- | --- |
| Subjects (n) | 28 | 10 | 52 | 20 |
| Males (n) | 8 | 2 | 8 | 10 |
| Females (n) | 20 | 8 | 44 | 18 |
| Age (years, mean ± SD) | 56.8 ± 10.4 | 62.9 ± 6.1 | 42.1 ± 10.6 | 45.2 ± 12.9 |

**Supplemental Table 3. Cerebrospinal fluid (CSF) sample patient demographics.**

| Variable | Primary progressive MS  (PPMS) | Relapsing remitting MS  (RRMS) | Other non- MS disorders * |
| --- | --- | --- | --- |
| Subjects (n) | 6 | 6 | 5 |
| Males (n) | 5 | 4 | 0 |
| Females (n) | 1 | 2 | 5 |
| Age (years, mean ± SD) | 56.5 ± 14.8 | 54.5 ± 23.5 | 65.6 ± 21.9 |

* We included the following Other non-MS disorders with the number of cases in parenthesis: Clinically Isolated Syndrome (1), Systemic lupus erythematosus (1), Cavernous hemangioma (1), Von Willebrand's Disease (1), Coronary Artery Disease (1).

**Supplemental Table 4. Antibodies and reagents used in the study.**

| **Antibodies used for Immunocytochemistry** | | | |  |
| --- | --- | --- | --- | --- |
| **Name** | **Company** | **Cat#** | **Conc** | **dilution** |
| Goat anti-human IgG (H+L) | Vector Lab | AI-3000 | 1.5mg/ml | 1:400 dilution |
| Mouse anti-Tuj1 | Sigma | 05-559-I | 0.5mg/ml | 1:400 dilution |
| Mouse anti-GFAP | Sigma | G3893 | 1 mg/ml | 1:400 dilution |
| Rabbit anti-human C1q | DAKO | A0136 | 7mg/ml | 1:400 dilution |
| Rabbit anti-human C3b | Mybiosource | MBS2559642 | 0.4mg/ml | 1:400 dilution |
| rabbit anti-C5b9 | Abcam | Ab55811 | 5mg/ml | 1:500 dilution |
| Rabbit Caspase3 | Sigma | C8487-25ul | 0.5 mg/ml | 1:200 dilution |
| Mouse anti-human IgG(H+L) | Invitrogen | SA1-35468 | 1.2 mg/ml | 1:200 dilution |
| Mouse anti-IgG3 | Sigma | SAB4200759-100ul | 1mg/ml | 1:200 dilution |
| Mouse anti-IgG1-Fc specific | Sigma | SAB4200788-100ul | 1mg/ml | 1:200 dilution |
| Mouse anti-MAP2 | Millipore | MAB378 | 0.5 mg/ml | 1:200 dilution |
| Goat anti-GFAP | Novusbio | NB100-53809 | 0.5 mg/ml | 1:300 dilution |
| Donkey anti-Goat AF 647 | Invitrogen | A21447 | 1 mg/ml | 1:400 dilution |
| Donkey anti-Goat AF 488 | Invitrogen | A11055 | 1 mg/ml | 1:400 dilution |
| Donkey anti-Goat AF 594 | Invitrogen | A11058 | 1 mg/ml | 1:400 dilution |
| Donkey anti-Rabbit AF 594 | Invitrogen | A21207 | 1 mg/ml | 1:400 dilution |
| Donkey anti-Rabbit AF 488 | Invitrogen | A21206 | 1 mg/ml | 1:400 dilution |
| Donkey anti-mouse AF 594 | Invitrogen | A21203 | 1 mg/ml | 1:400 dilution |
| Donkey anti-mouse AF 488 | Invitrogen | A21202 | 1 mg/ml | 1:400 dilution |
| Goat anti-mouse AF 568 | Abcam | Ab175473 | 1mg/ml | 1:400 dilution |
| Hoechst 33258 | Invitrogen | 62249 | 20 mM | 1:1000 dilution |
| Anti-fade mounting media | SouthernBiotech | 0100-20 |  |  |
|  |  |  |  |  |
| **Antibodies as an inhibitor of MS A-FT** | | | |  |
| Mouse anti-CD20 | R&D system | MAB4225 | 1mg/ml |  |
| Goat anti-human IgG-Fc | Rockland | 609-1103 | 5 mg/ml |  |
|  |  |  |  |  |
| **Inhibitor for MS A-FT neurotoxicity** | | |  |  |
| Pan caspase inhibitor Z-VAD-FMK | Promega | G7231 |  |  |
| Mitoxantrone | Cayman Chemical | 14842 |  |  |
| C1q-Depleted NHS | Complement Technology | A300_C1q-Dep |  |  |
|  |  |  |  |  |
| **Digestion of IgG and breakdown aggregates** | | |  |  |
| IdeS | Genovis | A0-FR1-008 |  |  |
| 8M Urea treatment | Sigma | 51457 |  |  |

**Supplemental Figure 1: Top three networks obtained from proteomic data of A-FT**


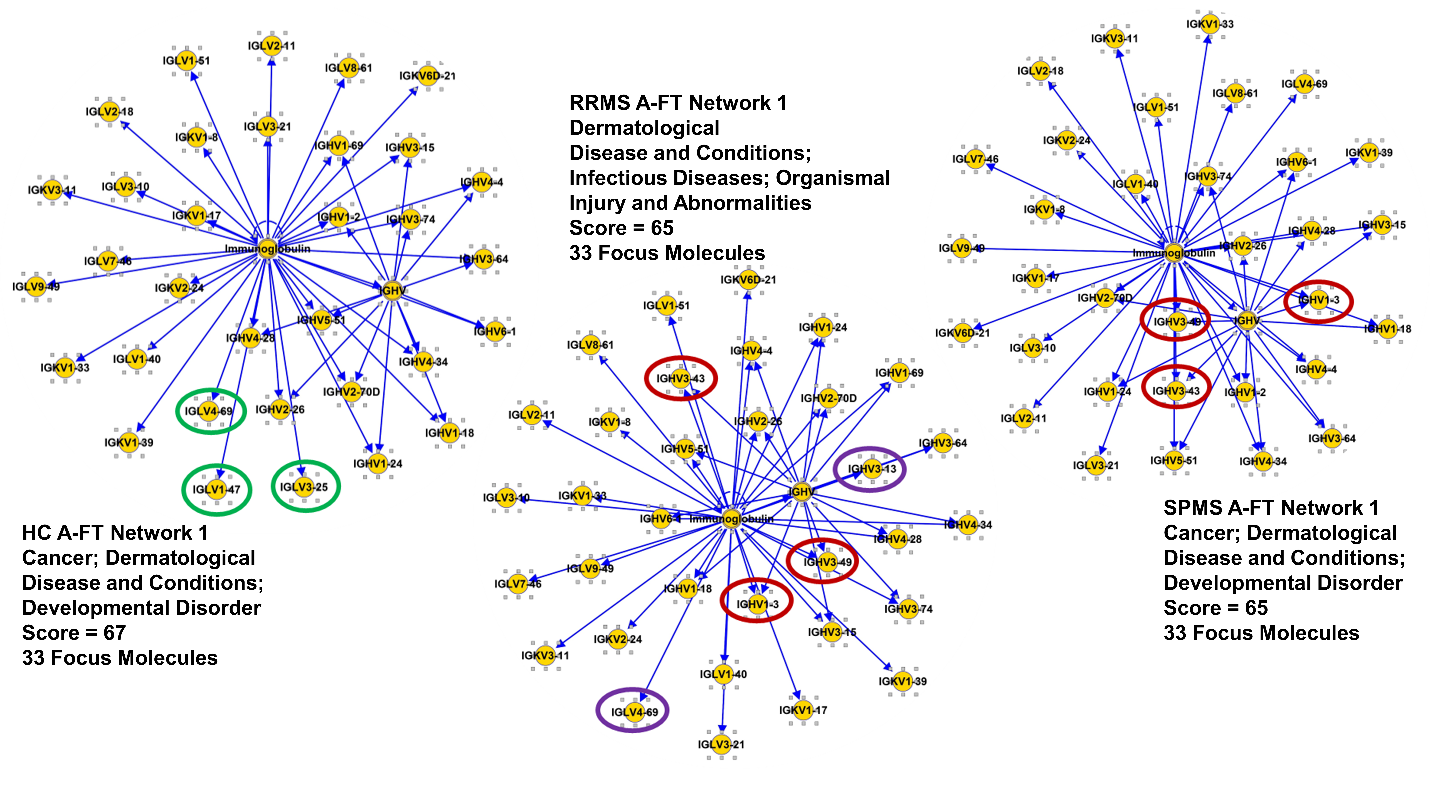


**Supplemental Figure 1**. Ingenuity Pathway Analysis (IPA) algorithms were used to generate the top three network interactomes from Comparison Analyses following Core Analyses of HC A-FT (left), RRMS A-FT (middle), and SPMS A-FT (right) proteomes. The top networks for each dataset (HC, RRMS, SPMS) are shown. Proteins in the networks identified from the proteomic data are shaded gold. Solid blue lines indicate direct, documented connections between proteins identified in the proteomic data set. Network scores are calculated from the right-tailed Fisher's Exact Test and provide a level of significance. Focus molecules are “seeds” that generate the basis for the networks. Green circles in the HC A-FT network indicate molecules only found in that network. Red circles in the RRMS and SPMS A-FT networks indicate molecules common to those networks, but not in the HC A-FT. Purple circles indicate molecules unique to the RRMS A-FT network.

**Supplemental Figure 2:** **Complement-based networks obtained from IPA**


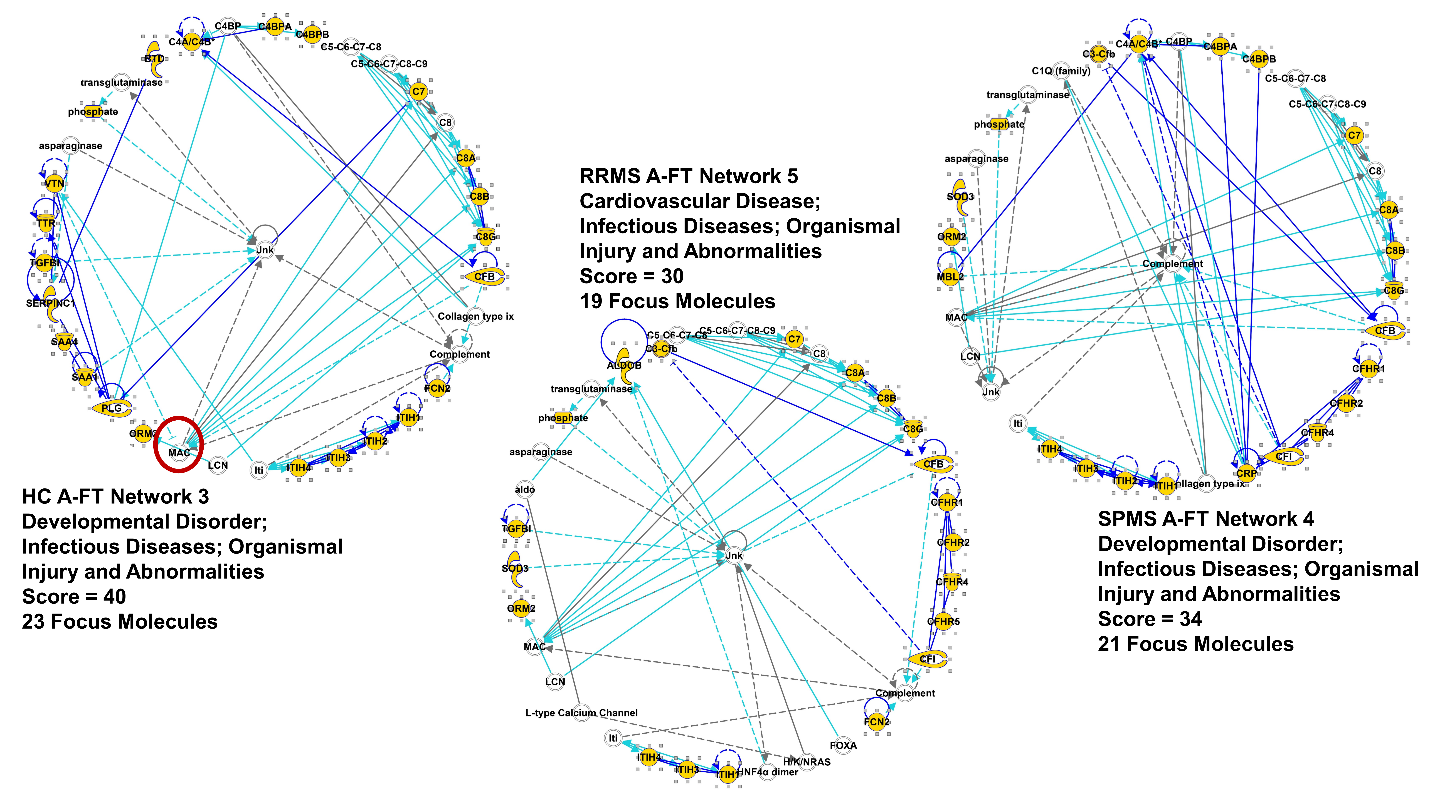


**Supplemental Figure 2** Complement-based networks obtained from IPA. HC A-FT (left), RRMS A-FT (middle), and SPMS A-FT (right) are presented in radial layout to emphasize the central main nodes (“JNK” for HC and RRMS, and “Complement” for SPMS) and connectivity of the membrane attack complex (“MAC”) to its various complement components. Proteins in the networks identified from the proteomic data are shaded gold. Solid blue lines indicate direct, documented connections between proteins identified in the proteomic data set. Broken lines represent indirect connections arising from reasoned speculation, or via known intermediaries, from the Ingenuity knowledgebase. Light blue/turquoise lines connect identified proteins within the network with proteins not part of the array. The red circle in the HC A-FT network highlights the potential MAC inhibition by vitronectin (VTN).

**Supplemental Figure 3. Confocal images of cells treated with healthy control (HC) A-FT and time course of MS A-FT treated cells**


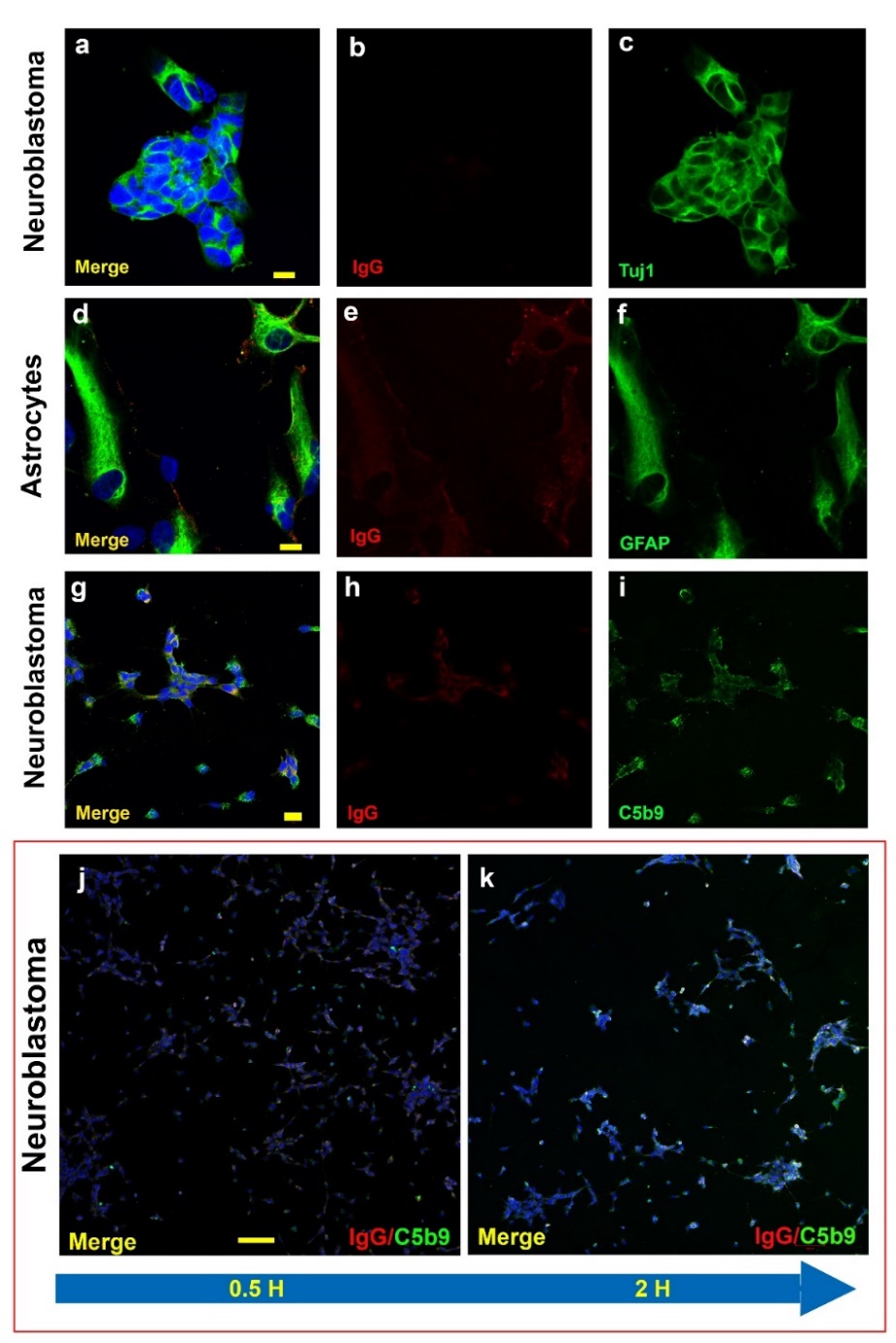


**Supplemental Figure 3. Confocal images of cells treated with healthy control (HC) A-FT and time course of MS A-FT treated cells.** Neuroblastoma SH-SY5Y cells (**a-c**) and astrocytes (**d-f**) were treated with HC A-FT for 2 hours and stained for IgG and cell-specific markers. There were few IgG-positive cells in HC A-FT treated cultures. **g-i**. SH-SY5Y cells were treated with HC A-FT for 2 hours and stained with C5b9 (the membrane attack complex) and IgG antibodies. There were few C5b9 positive cells at 2 hours of treatment. **j-k**. Low magnification view (10x) of SH-SY5Y cells treated with MS A-FT for 0.5 hours (j) and 2 hours (2k). Cells were co-stained for IgG (red) and C5b9 (green) antibodies. By visual assessment, there appeared to have fewer cells at 2 hours compared with 0.5 hours of treatment. Scale bar, 20 µm (**a-f**), 10 µm (**g-i**), 100 µm (**j-k**).

**Supplemental Figure 4. Confocal images for IgG and Caspase-3 in three types of cells treated with HC A-FT**


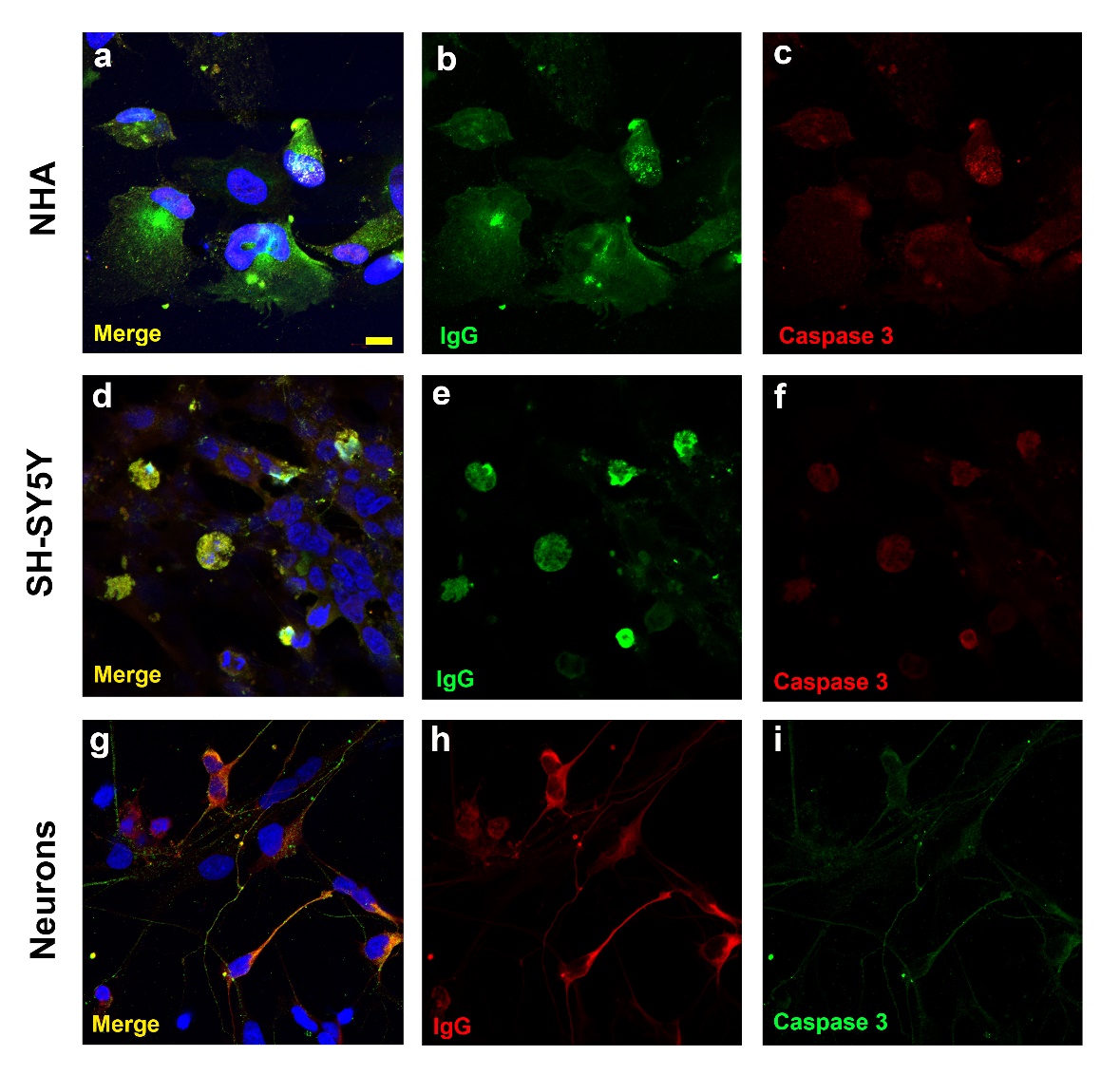


**Supplemental Figure 4.** **Confocal images for IgG and Caspase-3 in three types of cells treated with HC A-FT**. Normal human astrocytes (NHA) (**a-c**), neuroblastoma SH-SY5Y (**d-f**), and primary human neurons (**g-i**) were treated with HC A-FT for 2 hours and stained with IgG and the apoptosis marker Caspase-3 antibodies. IgG antibodies are present on the cell surface in all three cell types. But there were few Caspase-3 positive cells detected, indicating a lack of apoptosis. Bar, 10 µm (a-i).
